# Particle-Breaking Hartree–Fock Theory for Open Molecular Systems

**DOI:** 10.1021/acs.jpca.2c07686

**Published:** 2023-01-31

**Authors:** Regina Matveeva, Sarai Dery Folkestad, Ida-Marie Høyvik

**Affiliations:** Department of Chemistry, The Norwegian University of Science and Technology, Trondheim7491, Norway

## Abstract

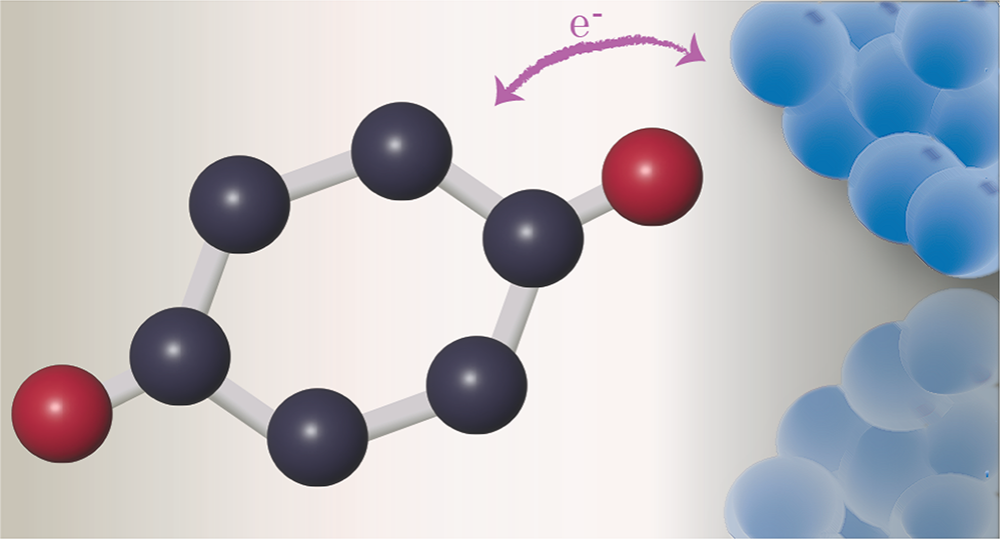

In this work we present the particle-breaking Hartree–Fock (PBHF) model which is a mean-field approach to open molecular systems. The interaction of a system with the environment is parametrized through a particle-breaking term in the molecular Hamiltonian. The PBHF wave function is constructed through an exponential unitary transformation of a Slater determinant with a given number of electrons. We consider only the closed-shell formalism. The parametrization results in a linear combination of Slater determinants with different numbers of electrons, i.e., the PBHF wave function is not an eigenfunction of the number operator. As a result, the density matrix may have fractional occupations which are, because of the unitary parametrization, always between 0.0 and 2.0. The occupations are optimized simultaneously with the orbitals, using the trust-region optimization procedure. In the limit of a particle-conserving Hamiltonian, the PBHF optimization will converge to a standard Hartree–Fock wave function. We show that the average number of electrons may be decreased or increased depending on whether the particle-breaking term affects occupied or virtual orbitals.

## 1 Introduction

In molecular electronic-structure theory we usually consider molecules for which the number of electrons is conserved, even when a molecule is interacting with an environment. However, in some cases it may be beneficial to view molecules as open with respect to the flow of electrons or electronic charge. An example of open systems are molecular junctions,^1−3^ in which a single molecule acts as a bridge for electron transport between some contact points. Furthermore, electron fluctuation across regions should be allowed in embedding and QM/MM theories.^4−10^ Hence, an explicit treatment of the electronic-structure for open molecular systems is called for.

In this paper, we present the particle-breaking Hartree–Fock (PBHF) model for open molecular systems where the electronic Hamiltonian does not commute with the number operator. The Hamiltonian contains a particle-breaking term which parametrizes the interaction of the molecule with the environment, e.g., the environment may act as a source or drain of electrons. A particle-breaking state is obtained using a unitary transformation of a particle-conserving reference Slater determinant. Here, we present a closed-shell formulation, but an extension to open-shells is possible. The unitary transformation is generated through an exponential parametrization using two-body creation and annihilation operators. This yields a linear combination of closed-shell Slater determinants with different number of electrons. As a consequence, the density matrix for the particle-breaking state may have fractional occupations. Due to the exponential parametrization, the occupations are between 0.0 and 2.0 for any choice of parameters. An unconstrained optimization over these parameters may therefore be carried out, while simultaneously optimizing the orbitals. We use a trust-region^11^ based optimization approach, as this is seen to be reliable for similar wave function optimization problems.^12−15^ For a particle-conserving Hamiltonian, the wave function optimization will converge to the standard (particle-conserving) Hartree–Fock (HF) solution with integer occupations. However, unlike HF, the PBHF model can change the number of electrons in a system by passing through fractionally occupied states.

Since their introduction,^16,17^ fractional occupations have been used extensively to include electron correlation effects. They have been applied in studies of open-shell systems,^18,19^ for problems involving orbital degeneracies,^20−22^ to describe bond dissociation processes,^23−29^ for chemical reactivity,^30^ to provide spherically symmetric initial atomic densities,^31,32^ in calculations of excitation and ionization energies,^33,34^ and for photochemistry and dynamics.^35−38^ In PBHF, fractional occupations are purely a consequence of the particle-breaking nature of the wave function. Hence, PBHF is a mean-field particle-breaking state.

The PBHF model is similar to the Hartree–Fock–Bogoliubov (HFB) model, which is widely used^39−42^ in physics. However, we note that PBHF and HFB are introduced to serve different purposes. HFB unifies the description of mean-field and pairing correlations in terms of quasiparticles.^43^ The HFB ground state is a vacuum with respect to the quasi-particles. The PBHF ground state, on the other hand, can be viewed as an HFB ground state filled with quasi-particles, i.e., an HFB excited state.^44^ As the PBHF wave function is constructed using a unitary transformation, no additional constraints are required to ensure normalization of the wave function, which is not the case for HFB. The PBHF expectation value of a particle-conserving Hamiltonian yields an energy term identical to the so-called pairing energy of HFB. For molecules, this term is positive due to electrons repelling each other, and any fractional occupation will raise the energy. Hence, for a particle-conserving molecular Hamiltonian, both HFB and PBHF will reduce to an HF state. Despite this, several approaches^45−50^ for describing strong correlation in particle-conserving molecular systems have been inspired by HFB. A notable example is the constrained pairing-mean-field theory^51^ (CPMFT), which is built upon ζ-HFB where the pairing energy term is scaled by a factor ζ.^50^ When ζ is negative, the pairing energy term is attractive for electrons, and consequently there is an energy minimum with fractional occupations for a standard electron-conserving Hamiltonian. However, it should be noted that the two-particle density matrix in CPMFT is not *N*-representable, as the energy functional is no longer associated with a wave function. Although CPMFT breaks conservation in the number of electrons, it is restored on average using a Lagrange multiplier.^52−54^

Whereas the electronic-structure theory for closed molecular systems is well-established, this is not the case for open systems. This is the motivation for developing the PBHF model, which may be viewed as a generalization of the HF model to open systems. By preserving the connection to electronic wave function theory for closed systems, we may build up an analogous framework for open molecular systems. Hence, PBHF will be the cornerstone for the development of correlated models as well as response theory.

The paper is organized as follows. In Section 2, we introduce the PBHF wave function and illustrate important aspects of the model such as electron spread, size extensivity, active space version, and optimization details. In Section 4, we provide results demonstrating the behavior of PBHF for both particle-conserving and particle-breaking Hamiltonians. In particular, we present results showing how the orbital occupations change with the strength of the particle-breaking term, and a toy model of a molecule interacting with an environment which has certain source/drain characteristics is presented. Finally, in Section 5, we provide a summary and concluding remarks.

## 2 Theory

In this section, we describe the PBHF wave function. We start by setting up the particle-breaking Hamiltonian and introduce the wave function parametrization. A minimal example is used to demonstrate the features of the PBHF wave function. Finally, we provide details for the wave function optimization.

### 2.1 The Hamiltonian

We consider a Hamiltonian which consists of the standard nonrelativistic molecular electronic Hamiltonian, *H*_mol_, and a particle-breaking contribution, *H*_pb_,
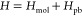
1The molecular electronic Hamiltonian in the orthonormal molecular orbital (MO) basis is given as
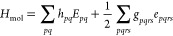
2where the nuclear repulsion has been excluded, and the one- and two-electron integrals are defined as
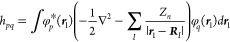
3
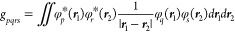
4*Z*_*n*_ is the nuclear charge, ***r***_*n*_ and ***R***_*n*_ are the electronic and the nuclear coordinates, and {φ_*p*_} are the MOs. The singlet one-electron and two-electron excitation operators in eq 2 are defined as
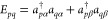
5
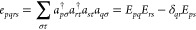
6

The particle-breaking Hamiltonian represents the interaction between the molecule and the environment, and it is here controlled through a single coupling parameter for each orbital, {λ_*p*_} (see Section 2.2 for details). Therefore, this parameter is a simple parametrization of the strength of the interaction between the molecule and a fictitious source or drain of electrons, as well as the ability of the source (drain) to donate (accept) electrons to (from) the molecule. The particle-breaking Hamiltonian is given by
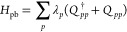
7where  and *Q*_*pp*_ are singlet closed-shell pair-creation and -annihilation operators. These are defined as
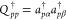
8
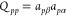
9

The particle-breaking Hamiltonian therefore does not commute with the number operator, *N* = *∑*_*p*_*E*_*pp*_,
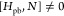
10Hence, approximations to the eigenfunctions of the Hamiltonian given in eq 1 should in general not be eigenfunctions of the number operator.

### 2.2 Particle-Breaking Hamiltonian

In this section we show how a Hamiltonian for a full system reduces to a particle-breaking Hamiltonian when only a subsystem is of interest. We consider a system that consists of subsystems *A* and *B* which may or may not be covalently bonded to each other. We carry out an orbital space partitioning of the orbital space {*P*} into an orbital space {*p*} for subsystem *A* and an orbital space  for subsystem *B*, i.e., . The Hamiltonian can be divided into three parts: a part describing subsystem *A*, a part for subsystem *B*, and the part describing their interaction. In the following we consider only the interaction part of the Hamiltonian (*H*^int^). Since we aim at a closed-shell formalism for subsystem *A*, we limit our discussion to the two-electron part of the Hamiltonian,
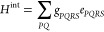
11where *P*, *Q*, *R*, *S* are general indices for the full system. Due to the closed-shell formalism, only terms containing two indices in each subsystem will be different from zero upon taking the expectation value. We get
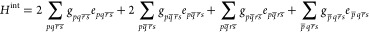
12where unbarred and barred indices refer to subsystem *A* and subsystem *B*, respectively. Assume that we are interested in subsystem *A* (target subsystem) whereas subsystem *B* is an environment and will not be treated explicitly. The first two terms of eq 12 represent standard two-electron interactions between the electrons in *A* and the electrons in *B*. We will not discuss these interactions further, as they are particle-conserving and may easily be included as done in existing multilevel approaches, e.g. in multilevel HF theory.^8^ The last two terms in eq 12 are particle-breaking for subsystem *A* and subsystem *B* (but particle-conserving for the full system). Below we show in detail how these two terms give rise to the simple particle-breaking Hamiltonian given in eq 7.

We insert the definition of the two-electron singlet excitation operator from eq 6 in the last two terms of eq 12 and use anticommutation rules to write,
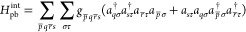
13Keeping only the closed-shell components, we obtain
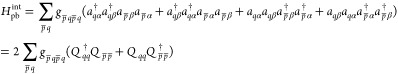
14

To see how an approximate particle-breaking interaction described by eq 14 arise, we consider the state, |Ψ^AB^⟩, for the full system
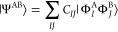
15where  represents a Slater determinant for the target subsystem and  is a Slater determinant for subsystem *B* (the environment). The determinants in the set  contain the same number of electrons, since |Ψ^AB^⟩ describes an overall closed system. However, the determinants  contain various numbers of electrons, as do . From eq 15, we generate an uncorrelated |Ψ^AB^⟩ by approximating the expansion coefficients as . The state |Ψ^AB^⟩ then can be rewritten as a product of states for *A* and *B*,
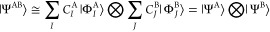
16Note that in this approximation, |Ψ^AB^⟩ is no longer particle-conserving. The effective particle-breaking operator for subsystem *A* is obtained by averaging  given in eq 14 with respect to the state |Ψ^B^⟩,
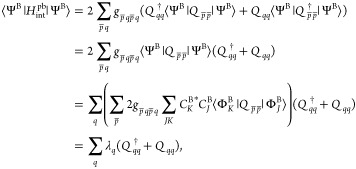
17where we used . Hence, the parameter λ_*q*_ contains information about the strength of the electronic interaction (through the two-electron integrals) and on the ability of the environment to acquire or donate electrons (through coefficients ). If Ψ^B^ is well-described by Slater determinants for the neutral state (i.e.,  for cationic and anionic Slater determinants), we obtain  and hence λ_*q*_ ≅ 0. We note that Ψ^B^ is never constructed, it is merely used to illustrate the origin of the simplest possible parametrization of the environment represented by λ. This derivation further shows that physical values of λ are small relative to contributions from the Hamiltonian of the target subsystem.

### 2.3 Wave Function Parametrization

In this section we describe the parametrization of a particle-breaking wave function for a system interacting with the environment (system *A* of Section 2.2). The particle-breaking approach presented here is restricted to closed-shell states, and is based on a unitary exponential parametrization of a closed-shell reference Slater determinant |Φ⟩,
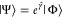
18where the antihermitian operator  is defined through the singlet pair-creation and -annihilation operators given in eqs 8 and 9
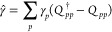
19

We have used a hat-notation to clearly distinguish between the operator  and the parameters {γ_*p*_}. The singlet spin-symmetry of the closed-shell reference state |Φ⟩ is preserved by the singlet pair-creation and -annihilation operators, and hence |Ψ⟩ of eq 18 is of singlet spin-symmetry. The summation over *p* in eq 19 may run over all orbitals or over a subset of occupied and/or virtual orbitals in the reference determinant (see Section 2.6). The resulting wave function |Ψ⟩ is a linear combination of determinants which contain various number of electrons. Hence, |Ψ⟩ is not an eigenfunction of the number operator. Furthermore, since the exponential transformation of the determinant |Φ⟩ in eq 18 is unitary (due to ), |Ψ⟩ is normalized for any choice of parameters {γ_*p*_} (provided that the reference determinant |Φ⟩ is normalized). We note that the PBHF wave function resembles a multiconfigurational self-consistent field (MCSCF) wave function.^55,56^ However, compared to the MCSCF configuration expansion coefficients, coefficients that determine the weights of single determinants in the PBHF wave function are not determined directly but through the parameters {γ_*p*_}.

### 2.4 The Energy Expression

The electronic energy of the parametrized state is given by the expectation value of the Hamiltonian in eq 1,
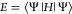
20The PBHF wave function is obtained by minimizing the energy, *E*, with respect to orbital rotations and wave function parameters {γ_*p*_}. In Section 2.9, we outline the trust-region optimization procedure used to find the energy minimum.

When working with the expectation value of operators with respect to the state |Ψ⟩ it is more convenient to work with |Φ⟩ and transformed operators, i.e., for eq 20 we use
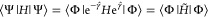
21The transformed Hamiltonian operator, , is expressed in terms of transformed creation and annihilation operators,
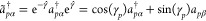
22
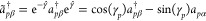
23where the Baker–Campbell–Hausdorff expansion and Taylor series of trigonometric functions are used to obtain the last equalities. Note that the singlet spin-symmetry of the reference determinant is preserved due to the definition of  in terms of singlet pair-creation and -annihilation operators (eq 19). The transformed singlet excitation operator  can be found by transforming the creation and annihilation operators of eq 5 according to eqs 22 and 23, and results in the following expression
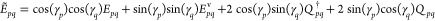
24

We have introduced a virtual singlet excitation operator 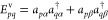
25which is connected to the standard singlet excitation operator by . The open-shell singlet pair-creation and pair-annihilation operators are defined as
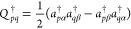
26
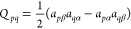
27The prefactor 1/2 is used to ensure that  and *Q*_*pq*_ reduce to  and *Q*_*pp*_ of eqs 8 and 9 when *p* = *q*.  and *Q*_*pp*_ were defined without prefactors for simplicity.

We may now set up the expressions for the one- and two-electron density matrices. The one-electron density matrix elements  are given by
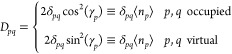
28From eq 28 we see that occupation numbers, ⟨*n*_*p*_⟩, may be fractional, depending on γ_*p*_. Hence, we use the term *occupation angles* for the wave function parameters {γ_*p*_}. Note that “occupied” and “virtual” refer to whether an orbital is occupied or virtual in the reference determinant |Φ⟩. Only the particle-conserving terms of  contribute to the expectation value  since |Φ⟩ is an *N*-electron Slater determinant. Furthermore, the occupied block of the density is given by the *E*_*pq*_ term in eq 24, whereas the virtual block is determined by the  term. For particle-breaking terms, we introduce the so-called *pairing* density matrix, η, whose elements are given as
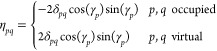
29

Using the definition for the standard and pairing density matrix elements given in eqs 28 and 29, we obtain following expression for the energy,
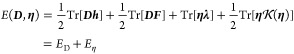
30where we have defined  and . The Fock matrix, ***F***, in the MO basis is given by
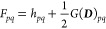
31where *G*(***D***)_*pq*_ = *∑*_*r*_(2*g*_*pqrr*_ – *g*_*prrq*_)*D*_*rr*_ is the two-electron interaction matrix. The MO one- and two-electron integrals *h*_*pq*_ and *g*_*pqrs*_ are defined in eq 3. The matrix **λ** is diagonal and contains the parameters λ_*p*_ of the particle-breaking Hamiltonian (eq 7). In eq 30, we have also introduced the matrix with elements
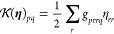
32If the particle-breaking term of the Hamiltonian dominates due to large λ_*p*_ values, the energy minimization in eq 30 will yield pairing density matrix elements of −2.0. This is obtained when the average orbital occupation is 1.0, i.e., for a half-filled orbital φ_*p*_.

### 2.5 Electron Spread of the Wave Function

The average number of electrons is given by the expectation value of the number operator *∑*_*p*_*E*_*pp*_, and for the state |Ψ⟩ this is
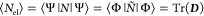
33where  is the number operator transformed using eqs 22 and 23.

The spread in the number of electrons is given by
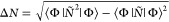
34If the Hamiltonian is particle-conserving, the PBHF optimization will yield a HF wave function. The spread in the number of electrons for such a state is zero. Using the definition of the transformed singlet operator  given in eq 24, we obtain
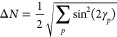
35From eq 35 it can be seen that Δ*N* = 0 when the parameters {γ_*p*_} are integer multiples of , i.e., all orbitals must be either doubly occupied or empty for the state  to be an eigenfunction of the number operator. This emphasizes that the fractional occupations in the density matrix are purely a consequence of the particle-breaking nature of the wave function.

### 2.6 Active Space Optimization of γ

Optimizing the γ-parameters for all orbitals may lead to slow convergence, since small variations in occupations close to 2.0 (for the occupied) or 0.0 (for the virtual) will only minimally affect the energy. Therefore, it may be beneficial to restrict the particle-breaking properties to certain orbitals, e.g., excluding core orbitals and high-lying virtual orbitals. Such an active space formulation is obtained by restricting the summation over *p* in eq 19 to a chosen subset of orbitals. This is equivalent to setting occupation angles γ_*p*_ to zero for orbitals whose occupation is unchanged relative to the reference determinant |Φ⟩. As a consequence, the one-electron density matrix elements given in eq 28 will be 2.0 (for the inactive occupied) and 0.0 (for the inactive virtual), whereas the average occupation of the active orbitals is defined in eq 28. For the pairing density matrix, the diagonals referring to the inactive space will be 0.0, while the elements for the active space are given by eq 29.

### 2.7 Size Extensivity

Considering two non-interacting systems, *a* and *b*, we may write
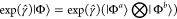
36using the size extensivity of the Slater determinant. The operator  contains only diagonal elements of the pair-creation and -annihilation operators, we may therefore write
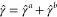
37where , because
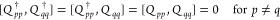
38Hence, we may factorize the exponential according to
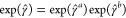
39and upon insertion into eq 36, we get
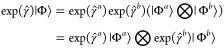
40establishing the size extensivity of the PBHF wave function.

### 2.8 Example: H_2_ in a Minimal Basis

In the minimal basis, the H_2_ molecule has two orbitals which we denote φ_*i*_ and φ_*a*_. We consider a reference determinant in which φ_*i*_ is doubly occupied
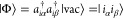
41The PBHF state is generated through , where
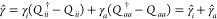
42

We obtain the following explicit expression for the PBHF state,
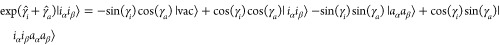
43

The PBHF state is a linear combination of the possible zero-, two-, and four-electron determinants with expansion coefficients given by trigonometric functions of the occupation angles. For arbitrary choices of γ_*i*_ and γ_*a*_, the PBHF state is not an eigenfunction of the number operator. We may, however, choose parameters for which an eigenfunction of *N* is obtained. For γ_*i*_ = γ_*a*_ = 0, the wave function reduces to the reference determinant |*i*_α_*i*_β_⟩, while for  we get −|*a*_α_*a*_β_⟩. It is not possible to choose parameters such that a linear combination of |*i*_α_*i*_β_⟩ and |*a*_α_*a*_β_⟩ (a correlated state) appears. This reflects the fact that the fractional occupations result from an average over determinants with different number of electrons. Hence, fractional occupations only occur when the state is not an eigenfunction of the number operator.

### 2.9 Optimization of the Wave Function

To optimize the wave function we use
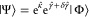
44

The changes in the occupation angles are generated by , and  is an anti-Hermitian orbital-rotation operator
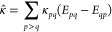
45where *E*_*pq*_ is defined in eq 5 and **κ** is an antisymmetric matrix (**κ**^*T*^ = −**κ**). Hence, normalization of the orbitals is preserved since  is a unitary operator. The variations of the standard and paring density matrices may be written as
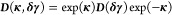
46
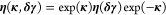
47

We collect the optimization parameters in the vector
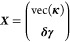
48where vec(**κ**) vectorizes **κ** by stacking its columns. We set up a quadratic model for the energy, expanded around ***X*** = 0,
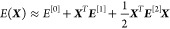
49where *E*^[0]^ is the energy value, ***E***^[1]^ is the gradient, and ***E***^[2]^ is the Hessian, all evaluated at the expansion point,
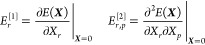
50

We minimize the energy functional using a trust-region optimization procedure.^11^ Here, the trust-region step constraint is defined through the Lagrangian,
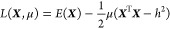
51

By differentiating eq 51 with respect to ***X*** and setting the result equal to zero, we obtain a level-shifted Newton step equation,
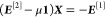
52

This equation is solved in a reduced space by using a Davidson solver,^57^ and hence we need the linear transformation of the Hessian on a vector, **σ** = ***E***^[2]^***X***, rather than ***E***^[2]^ itself. ***E***^[1]^ and **σ** have parts referring to the orbital-rotation parameters (denoted by a superscript **κ**) and the changes in occupation angles (denoted by a superscript ***δγ***). The gradient is given by
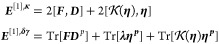
53where ***D***^*p*^ and **η**^***p***^ are matrices containing first derivatives of the standard and pairing densities with respect to the change in occupation angles. Note that there is no **λ** entering ***E***^[1],**κ**^, since **λ** is a matrix of fixed parameters. Elements of ***D***^*p*^ and **η**^***p***^ are given by
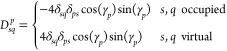
54
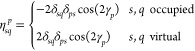
55For the linear transformation of the Hessian on the vector ***X*** we obtain
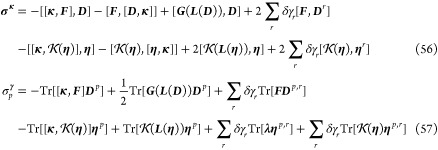
where
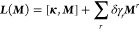
58and ***D***^*p*,*r*^ and **η**^*p*,*r*^ denote second derivatives of the standard and pairing densities, respectively. Elements of ***D***^*p*,*r*^ and **η**^*p*,*r*^ are given by
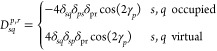
59
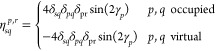
60

## 3 Computational Details

The PBHF framework and trust-region solver (see ref (58) for details) are implemented in a development version of the *e*^*T*^ program.^59^ A convergence threshold of 10^–7^ on the energy gradient (maximum norm of gradient) is used. The geometry of 1,4-benzenedithiol is taken from ref (60), and the geometries from the Thiel set are obtained from ref (61). All molecular structures are visualized using the UCSF Chimera software package.^62^ In the calculations with a particle-breaking Hamiltonian (Sections 4.2, 4.3, and 4.4), we apply the coupling parameters λ_HOMO_ and λ_LUMO_, where HOMO and LUMO refer to the orbitals in the reference determinant. All other coupling parameters are set to 0.0.

## 4 Results

In this section we present calculations illustrating the features of the PBHF model, both for particle-conserving and particle-breaking Hamiltonians. For the latter, we show how the strength of particle-breaking term affects the average occupation of orbitals in the wave function. Finally, we present a toy model of a molecule interacting with an environment which acts as source or drain of electrons.

### 4.1 The PBHF Wave Function for a Particle-Conserving Hamiltonian

In the following, we demonstrate two simple features of the PBHF wave function: that an HF solution is found for a particle-conserving Hamiltonian, and that the number of electrons is determined automatically during the energy optimization. That is, the PBHF optimization for a particle-conserving Hamiltonian yields an HF solution that is an energy minimum—both with respect to the orbital rotation parameters and the number of electrons.

PBHF calculations for the standard (particle-conserving) molecular-electronic Hamiltonian (see eq 2) were performed for the Thiel set of molecules using the cc-pVDZ basis.^63^ The initial charges of the molecules were chosen to be either +2 or −2. The results, in terms of the final charges and energies, are presented in Table 1. The final spread in the number of electrons Δ*N* is 0.0, indicating that the state is particle-conserving and the final energies are identical to the HF energies. In standard HF optimizations, the charge of the system cannot change. The resulting state will be an energy minimum with respect to orbital rotations, but not necessarily with respect to the number of electrons. For example, an HF optimization of a dication will converge to a dicationic HF state.

**Table 1:** PBHF Optimizations of the Thiel Set in the cc-pVDZ Basis with Initial Charges of ±2^a^

	Initial charge +2		Initial charge −2	
Molecule	Final charge	*E* (au)	Final charge	*E* (au)
Acetamide	0	–207.994496	0	–207.994496
Acetone	0	–191.975717	–2	–191.461754
Adenine	0	–464.557567	–2	–464.133718
Benzene	0	–230.721819	0	–230.721819
Benzoquinone	0	–379.258633	–2	–379.074369
Butadiene	0	–154.934104	0	–154.934104
Cyclopentadiene	0	–192.807780	–2	–192.360706
Cyclopropene	0	–115.832416	0	–115.832416
Cytosine	0	–392.649105	–2	–392.243957
Ethene	0	–78.039848	0	–78.243957
Formaldehyde	0	–113.874485	0	–113.874485
Formamide	0	–168.946490	0	–168.946490
Furan	0	–228.642106	0	–228.642106
Hexatriene	0	–231.828790	0	–231.828790
Imidazole	0	–224.833866	0	–224.833866
Naphthalene	0	–383.383266	0	–383.383266
Norbornadiene	0	–269.383265	–2	–269.219327
Octatetraene	0	–308.723542	0	–308.723542
Propanamide	0	–247.033160	–2	–246.514511
Pyrazine	0	–262.701263	0	–262.701263
Pyridazine	0	–262.667250	0	–262.667250
Pyridine	0	–246.714175	0	–246.714175
Pyrimidine	0	–262.712209	0	–262.712209
Pyrrole	0	–208.827725	0	–208.827725
Tetrazine	0	–294.610442	–2	–294.321264
Thymine	0	–451.544092	–2	–451.161841
Triazine	0	–278.715573	0	–278.715573
Uracil	0	–412.502227	–2	–412.118681

a Final charges and energies in au are presented.

The results in Table 1 demonstrate that the PBHF optimization for molecules described by a standard particle-conserving molecular electronic Hamiltonian reduces to an HF solution. However, the final charge of the optimized PBHF state may be different from the initial charge. This can be seen for all the dications. However, for some of the dianions (acetone, adenine, benzoquinone, cyclopentadiene, cytosine, propanamide, tetrazine, thymine, and uracil) the PBHF optimization preserves the initial charge. Here the dianionic states are local minima with respect to both orbital rotation parameters and number of electrons. The dianionic states are higher in energy than the neutral ones, but optimization algorithms in general cannot distinguish between local and global minima.

### 4.2 The Strength of the Particle-Breaking Term

**Figure 1:**
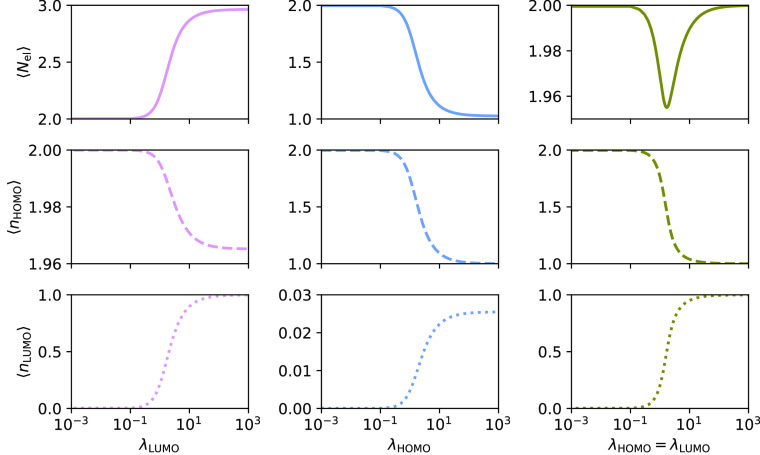
PBHF calculations for H_2_ using STO-3G. The effect on average number of electrons, ⟨*N*_el_⟩, the occupation of HOMO, ⟨*n*_HOMO_⟩, and LUMO, ⟨*n*_LUMO_⟩, with increasing values of λ_LUMO_ (left), λ_HOMO_ (middle), and both (right). Please note the different scales of the ordinate axes.

To illustrate how the particle-breaking term (eq 7) in the Hamiltonian affects the wave function, we consider the H_2_ molecule in a minimal basis. In Figure 1, we present the average number of electrons, ⟨*N*_el_⟩, and the occupations of φ_HOMO_ and φ_LUMO_ (HOMO and LUMO with respect to the reference determinant) as they change with increasing λ_HOMO_ and/or λ_LUMO_. Note that we also include λ values of unphysical magnitude (see Section 2.2) to demonstrate the behavior of the wave function in the limit of large λ.

From the left panel, we see that the primary effect of increasing λ_LUMO_ is to smoothly increase the occupancy of φ_LUMO_ up to 1.0, accompanied by a small relaxation in the occupation of the φ_HOMO_. This leads to a smooth increase in the expectation value of the number of electrons from 2.0 to almost 3.0. If we rather increase λ_HOMO_ (and keep λ_LUMO_ = 0.0, see the middle panel of Figure 1), we see a decrease in the occupation of φ_HOMO_ toward 1.0. The average number of electrons also decreases to almost 1.0, and small relaxation effects in the occupancy of φ_LUMO_ are observed. Hence, the effect of a λ parameter affecting an occupied orbital is to reduce its occupancy toward 1.0, whereas a λ parameter affecting a virtual orbital increases its occupancy toward 1.0. When both λ_HOMO_ and λ_LUMO_ increase (λ_HOMO_ = λ_LUMO_, see the right panel of Figure 1), we see, on average, the simultaneous draining of φ_HOMO_ and filling of φ_LUMO_. For intermediate values of λ, the number of electrons decreases slightly. For large values of λ, the expectation value of the number of electrons goes back toward 2.0.

We now examine the PBHF states for different λ values. We insert the computed wave function parameters, γ_HOMO_ and γ_LUMO_, into eq 43. First, we look at the state for λ_LUMO_ = 1000.0 and λ_HOMO_ = 0.0. The following approximate form of the wave function is obtained,
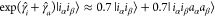
61

This PBHF state is approximately an equal mix of the determinant where φ_HOMO_ is doubly occupied and the determinant where both φ_HOMO_ and φ_LUMO_ are doubly occupied. The other two determinants, |vac⟩ and |*a*_α_*a*_β_⟩, also enter the wave function, but with weights smaller than 0.1. The electron spread (see eq 35) is Δ*N* = 0.5168.

Similarly, for λ_HOMO_ = 1000.0 and λ_LUMO_ = 0.0, we see that the wave function becomes
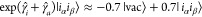
62i.e., approximately an equal mix of the vacuum state and the determinant where φ_HOMO_ is doubly occupied. Again, all four determinants in eq 43 enter the PBHF state, but the determinants |*a*_α_*a*_β_⟩ and |*i*_α_*i*_β_*a*_α_*a*_β_⟩ have weights smaller than 0.1. These small contributions give rise to the small fractional occupations of φ_LUMO_. The electron spread in this case is Δ*N* = 0.5124.

Finally, when λ_LUMO_ = λ_HOMO_ = 1000.0, the PBHF state is given as
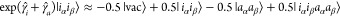
63and is an equal mix of all four determinants, and Δ*N* = 0.7071. These three approximate wave functions illustrate why ⟨*N*_el_⟩ in Figure 1 tends toward 3.0, 1.0, and 2.0 electrons (left, middle, and right panels, respectively) for large values of λ. Further increase of λ will not yield any significant changes in the wave function as the plateaus in ⟨*n*_*p*_⟩ are reached (see Figure 1). As described in Section 2.4, this will occur when the minimization of the energy is dominated by the diagonal elements of the pairing density matrix due to large values of λ.

**Figure 2:**
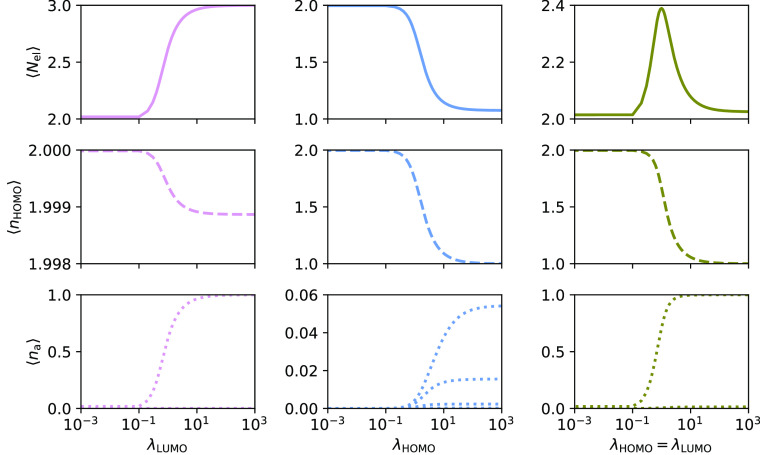
PBHF calculations for H_2_ using cc-pVDZ. The effect on average number of electrons, ⟨*N*_el_⟩, the occupation of HOMO, ⟨*n*_HOMO_⟩, and virtual orbitals, ⟨*n*_a_⟩, with increasing values of λ_LUMO_ (left), λ_HOMO_ (middle), and both (right). Please note that occupancies of other virtual orbitals in case λ_HOMO_ = λ_LUMO_ are not zero, although the corresponding λ parameters are, and note the different scales of the ordinate axes.

In Figure 2, we show the number of electrons, the occupations of φ_HOMO_, and the occupations of all the virtual orbitals, as they change with increasing λ_HOMO_ and/or λ_LUMO_ for H_2_ in the cc-pVDZ basis. The behavior of the average occupations and the number of electrons is the same for the two basis sets. However, in case of cc-pVDZ, relaxation effects are observed for several virtual orbitals for large values of λ_HOMO_. For λ_LUMO_ ≠ 0.0, the relaxation effects in the occupation of φ_HOMO_ are less pronounced. However, relaxation effects in the occupations of the other virtual orbitals are present and of the same order of magnitude as for φ_HOMO_.

**Figure 3:**
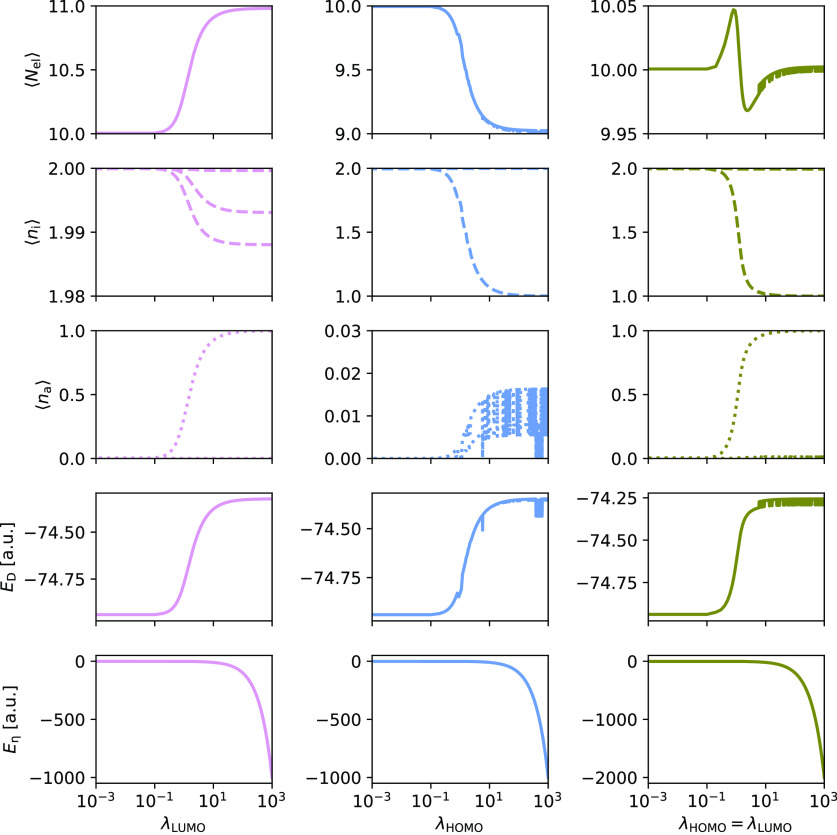
PBHF calculations for H_2_O using STO-3G. The effect on the number of electrons, ⟨*N*_el_⟩, the occupations of occupied orbitals, ⟨*n*_i_⟩, and virtual orbitals, ⟨*n*_a_⟩, as well as the energy contributions, *E*_D_ and *E*_η_, with increasing values of λ_LUMO_ (left), λ_HOMO_ (middle), and both (right).

**Figure 4:**
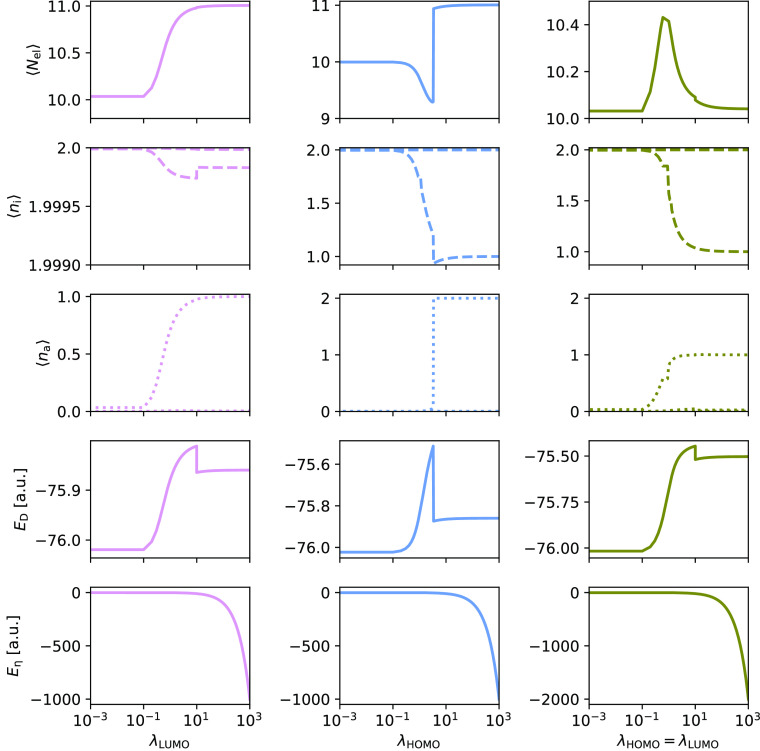
PBHF calculations for H_2_O using cc-pVQZ. The effect on the number of electrons, ⟨*N*_el_⟩, the occupations of occupied orbitals, ⟨*n*_i_⟩, and virtual orbitals, ⟨*n*_a_⟩, as well as the energy contributions, *E*_D_ and *E*_η_, with increasing values of λ_LUMO_ (left), λ_HOMO_ (middle), and both (right).

In Figures 3 and 4, we present the average number of electrons, the occupations of the occupied orbitals (φ_*i*_), the occupations of the virtual orbitals (φ_*a*_), and energy contributions as they change with increasing λ_HOMO_ and/or λ_LUMO_ for H_2_O in the STO-3G and cc-pVQZ basis sets, respectively. We plot *E*_D_ and *E*_η_ (see eq 30) separately, since the energy will be dominated by the term Tr[***ηλ***] in *E*_η_ as λ increases. For computational efficiency, each cc-pVQZ calculation for a given λ is restarted from the previous λ step.

The trends for H_2_O are, to a large extent, the same as for H_2_. However, there are two new features appearing for H_2_O. First, we observe convergence to different local minima. This can be seen for large λ_HOMO_ = λ_LUMO_ for both basis sets and for large λ_HOMO_ (λ_LUMO_ = 0.0) for STO-3G. The local minima have slightly different occupations as can be seen from Figures 3 and 4. Second, in Figure 4 we observe a discontinuity at around λ_HOMO_ = 10, related to a sudden change in the number of electrons. The system goes from having approximately nine electrons to approximately 11 electrons by filling a virtual orbital. This effect arises from a strong perturbation of only one orbital. In the PBHF framework a virtual orbital can become doubly occupied, if it is energetically favorable. The sudden occupation of a virtual orbital is coupled to a discontinuity in the occupation of φ_HOMO_ around ⟨*n*_HOMO_⟩ = 1.0.

### 4.3 Average Number of Electrons and Spread

**Figure 5:**
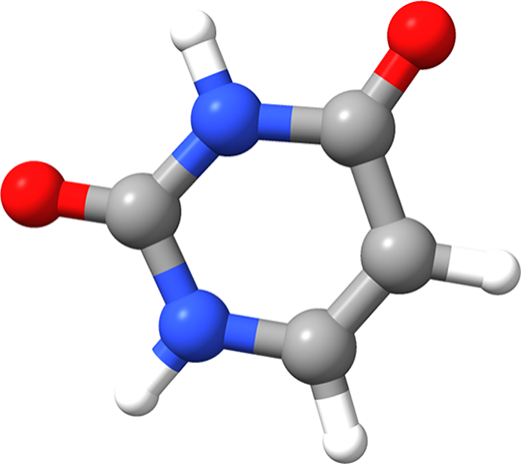
Uracil.

We now examine the behavior of the PBHF wave function for full and active space optimizations. We have performed STO-3G calculations on four molecular systems (ethene, pyrrole, formamide, and uracil) from the Thiel set using different combinations of λ_HOMO_ and λ_LUMO_ parameters. The results show the same trends for all the molecules, and, therefore, we present only uracil (see Figure 5) here. The average number of electrons, ⟨*N*_el_⟩, and the electron spread, Δ*N*, for full space as well various active space selections (symmetrically about HOMO and LUMO) for uracil are presented in Table 2. Results for ethene, pyrrole, and formamide are given in the Supporting Information.

**Table 2:** Average Number of Electrons, ⟨*N*_el_⟩, and Electron Spread, Δ*N*, from PBHF Calculations on Uracil Using STO-3G for Several λ Combinations and Various Active Spaces^a^

		Full space		2 active		4 active		6 active	
λ_HOMO_	λ_LUMO_	⟨*N*_el_⟩	Δ*N*	⟨*N*_el_⟩	Δ*N*	⟨*N*_el_⟩	Δ*N*	⟨*N*_el_⟩	Δ*N*
1	2	58.105	0.720	58.139	0.695	58.148	0.708	58.148	0.708
2	1	57.922	0.722	57.927	0.698	57.948	0.711	57.948	0.711
10	20	57.990	0.730	58.011	0.707	57.995	0.727	57.993	0.731
20	10	57.976	0.730	57.996	0.707	57.980	0.727	57.979	0.731
100	200	57.980	0.730	58.001	0.707	57.985	0.727	57.983	0.731
200	100	57.979	0.730	58.000	0.707	57.984	0.727	57.982	0.731

a Isolated neutral uracil has 58 electrons.

We first consider the results for the full space calculations. When λ_HOMO_ = 1.0 and λ_LUMO_ = 2.0, the average number of electrons is increased relative to the neutral isolated molecule. In contrast, when λ_HOMO_ = 2.0 and λ_LUMO_ = 1.0, the average number of electrons is reduced. Hence, we see that the environment acts as a source or drain of electrons when the strongest effect is on the LUMO or HOMO, respectively. When λ is increased by one or 2 orders of magnitude, the average number of electrons is slightly lower than that of the neutral molecule irrespective of which λ is largest. Also, the electron spread becomes constant.

We look at the occupations of the HOMO and LUMO orbitals for different orders of magnitude of λ parameter in more detail. When λ_HOMO_ = 1.0 and λ_LUMO_ = 2.0, the corresponding occupations are 1.226 and 0.906, respectively. For the combination λ_HOMO_ = 10.0 and λ_LUMO_ = 20.0, the corresponding occupations are 1.018 and 0.994. Similar occupations (⟨*n*_HOMO_⟩ = 1.000 and ⟨*n*_LUMO_⟩ = 0.998) are observed when λ_HOMO_ = 200.0 and λ_LUMO_ = 100.0. We see that for small values of the coupling parameter λ, the occupations of the two orbitals are unevenly affected. However, we note again, that in case of the larger λ values, the affected orbitals become half-filled. This supports the results presented in Table 2 and is consistent with the plateaus in the number of electrons and occupations seen in Figures 1–4. Hence, further increasing of λ will not affect the average number of electrons or spread.

When an active space rather than the full orbital space is used, some changes in the average number of electrons and spread are observed. For four (HOMO–1, HOMO, LUMO, and LUMO+1) and six (HOMO–2, HOMO–1, HOMO, LUMO, LUMO+1, and LUMO+2) active orbitals the qualitative picture is the same. When the interaction with HOMO dominates, the number of electrons is increased, whereas the number of electrons is decreased when the interaction with LUMO is strongest.

However, we note that in case of only two active orbitals (HOMO and LUMO) there is a slight increase in the average number of electrons rather than a decrease which is observed for the full space and other active space calculations. This indicates that an active space of two orbitals is insufficient to cover the effects of the full space and underlines the importance of relaxation effects. For two active orbitals, no relaxation effects in the occupation of other orbitals are possible.

### 4.4 A Toy Model: 1,4-Benzenedithiol

**Figure 6:**
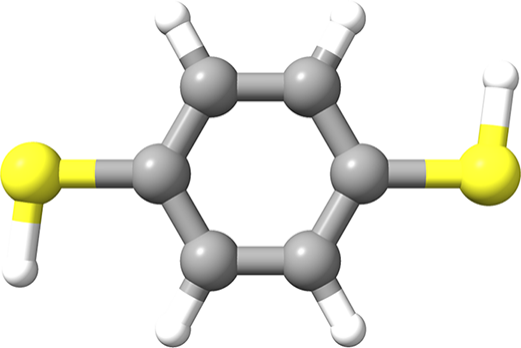
1,4-Benzenedithiol.

Here, a toy model for 1,4-benzenedithiol (see Figure 6) interacting with an environment which serves as a source or drain of electrons is presented. We use a simple form of the particle-breaking term in the Hamiltonian (eq 7). The effect of the environment is modeled by an energy, *W*, associated with either gaining or releasing an electron. Depending on *W* relative to the ionization energy (IE) and electron attachment energy (EA), the environment will act either as a source or drain for an electron with an associated probability (*p*). The coupling between 1,4-benzenedithiol and the environment is described by the electronic interaction energy *V*. Therefore, we use
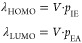
64where *p*_IE_ and *p*_EA_ are the probabilities of ionization and electron attachment, respectively. The probabilities (consistent with those presented in ref (64)) are
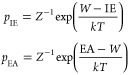
65where *Z*^–1^ is a normalization constant. For 1,4-benzenedithiol, we use the approximate values IE = 8.0 eV and EA = 2.0 eV, and in our simple model, we set *kT* = 1 eV^–1^. We present the change in the average number of electrons with respect to two values of *W*. First, *W* is chosen to be close to the electron attachment energy. Second, *W* is chosen to be close to the ionization energy. For each *W*, two strengths of electronic coupling to the environment are considered. In this picture, the electronic interaction between HOMO and the environment and between LUMO and the environment is taken to be identical. The results are listed in Table 3. We see that for *W* close to the electron attachment energy (*W* = 2.01 eV), the average number of electrons is 74.004 when *V* = 0.1 au and 74.273 when *V* = 1.0 au. Hence, on average, the molecule accepts some electronic charge from the environment. When *W* is close to the ionization energy (*W* = 7.99 eV), we see from Table 3 that the average number of electrons is 73.993 for *V* = 0.1 au and 73.601 for *V* = 1.0 au. Thus, the molecule donates some electronic charge to the environment.

**Table 3:** Average Number of Electrons and Spread for 1,4-Benzenedithiol for Different Combinations of λ_HOMO_ and λ_LUMO_^a^

*W* (eV)	V (au)	λ_HOMO_ (au)	λ_LUMO_ (au)	⟨*N*⟩	Δ*N*
2.01	0.1	1.257 × 10^–4^	4.969 × 10^–2^	74.004	0.0445
2.01	1.0	1.257 × 10^–3^	4.969 × 10^–1^	74.273	0.3491
7.99	0.1	4.969 × 10^–2^	1.257 × 10^–4^	73.993	0.0602
7.99	1.0	4.969 × 10^–1^	1.257 × 10^–3^	73.601	0.4046

a λ_HOMO_ and λ_LUMO_ are determined from a simple model of electron attachment and ionization probabilities relative to *W* and scaled by an electronic interaction strength *V* (eq 64). The calculations are carried out using the STO-3G basis set and all orbitals active. The neutral isolated molecule has 74 electrons.

## 5 Summary and Concluding Remarks

The particle-breaking Hartree–Fock (PBHF) model is a mean-field approach to open molecular systems. In this work, we have considered the closed-shell formalism, but an extension to open-shells is straightforward. Per definition, an open system interacts with an environment, and we parametrize this interaction through a particle-breaking term in the molecular Hamiltonian. The environment can act as source or drain of electrons. The strength of the coupling to the environment is controlled through a parameter λ for each orbital. The PBHF wave function is constructed through an exponential unitary transformation of a Slater determinant with a given number of electrons. This parametrization leads to a linear combination of Slater determinants with various number of electrons. Consequently, the resulting density matrix may have fractional occupations. In case of fractional occupations, the PBHF wave function is not an eigenfunction of the number operator. Since the parametrization is unitary, the occupations are always between 0.0 and 2.0. For a particle-conserving Hamiltonian, occupations will be 0.0 or 2.0, and the PBHF wave function becomes an eigenfunction of the number operator.

For the (particle-conserving) standard molecular electronic Hamiltonian, the PBHF optimization results in a HF solution. However, in the course of the optimization, the PBHF wave function may pass through fractional occupations before converging to an HF solution. Such an HF solution represents an energy minimum with respect to both orbital rotations and the number of electrons, i.e., if the number of electrons in the system before the optimization does not correspond to a minimum in this parameter space, the number of electrons will change.

For the particle-breaking Hamiltonian used in this work, the strength of λ for an occupied orbital determines the extent to which electrons are drained from that orbital. Similarly, the strength of λ for a virtual orbital determines the extent to which that orbital is filled. For large (unphysical) λ values, the average occupancy of the corresponding orbital will go toward half-filled. Orbitals with no coupling to the environment (λ = 0) may have slight changes in occupations due to relaxation effects.

The occupations are optimized simultaneously with the orbitals using a second-order trust-region optimization procedure. Small changes in orbital occupations may lead to a slow convergence of orbital rotations. Hence, freezing occupations which are close to 0.0 and 2.0 increases computational efficiency. This procedure is called active space PBHF optimization. It is important to include orbitals beyond those directly affected by λ, as relaxation effects generally occur and these effects are necessary to obtain (qualitatively) correct description.

Furthermore, we have illustrated—through our toy-model of 1,4-benzenedithiol—that depending on the electronic properties of the molecule and the environment, the molecule can acquire or donate electronic charge.

In conclusion, the PBHF model can describe molecular systems which are open to the flow of electrons or electronic charge to or from the surroundings, e.g., solvent or surface. Therefore, it has several potential applications, e.g., within the field of molecular electronics. For charge transport through molecular junctions, future developments of the PBHF model will be able to account for transfer of both integer and non-integer number of electrons. Furthermore, PBHF could be applied in embedding or QM/MM models by adding an adequately parametrized particle-breaking term to the Hamiltonian. This additional term will allow for partial charge exchange between regions.

## Supporting Information Available

Average number of electrons and electron spread for PBHF calculations on ethene, pyrrole and formamide are presented; see Section 4.3 for details (PDF)
