# Supplementary material for: Particle-Breaking Hartree–Fock Theory for Open Molecular Systems
Source: J Phys Chem A. 2023 Jan 31;127(5):1329–41. doi: 10.1021/acs.jpca.2c07686 (PMC9923758; doi:10.1021/acs.jpca.2c07686)
Supplement: Supplementary file 1 — jp2c07686_si_001.pdf [file jp2c07686_si_001.pdf]

# Supporting Information

# Particle-breaking Hartree-Fock Theory for Open Molecular Systems

Regina Matveeva‡, Sarai Dery Folkestad‡*, Ida-Marie Høyvik‡*

‡ Dept. of Chemistry, Norwegian University of Science and Technology,
7491 Trondheim, Norway

* E-Mail: Sarai Dery Folkestad – sarai.d.folkestad@ntnu.no
Ida-Marie Høyvik – ida-marie.hoyvik@ntnu.no

## Number of electrons and spread for ethene, pyrrole and formamide

Here we provide results for ethene, pyrrole and formamide as mentioned in Section 4.3 in the main manuscript.

**Table S1:** Average number of electrons, $\langle N_{el} \rangle$, and spread, $\Delta N$, from PBHF calculations on ethene using STO-3G for several $\lambda$ combinations and various active spaces. Isolated ethene has 16 electrons.

| | | Full space | | 2 active | | 4 active | | 6 active | |
|---|---|---|---|---|---|---|---|---|---|
| $\lambda_{\text{HOMO}}$ | $\lambda_{\text{LUMO}}$ | $\langle N_{el} \rangle$ | $\Delta N$ | $\langle N_{el} \rangle$ | $\Delta N$ | $\langle N_{el} \rangle$ | $\Delta N$ | $\langle N_{el} \rangle$ | $\Delta N$ |
| 1 | 2 | 16.197 | 0.689 | 16.197 | 0.674 | 16.198 | 0.675 | 16.198 | 0.675 |
| 2 | 1 | 15.803 | 0.677 | 15.798 | 0.673 | 15.803 | 0.675 | 15.803 | 0.675 |
| 10 | 20 | 16.008 | 0.710 | 16.008 | 0.707 | 16.008 | 0.707 | 15.993 | 0.720 |
| 20 | 10 | 15.991 | 0.710 | 15.992 | 0.707 | 15.992 | 0.707 | 15.978 | 0.717 |
| 100 | 200 | 15.971 | 0.721 | 16.001 | 0.707 | 16.001 | 0.707 | 15.973 | 0.720 |
| 200 | 100 | 15.998 | 0.710 | 15.999 | 0.707 | 15.999 | 0.707 | 15.998 | 0.710 |

**Table S2:** Average number of electrons, $\langle N_{el} \rangle$, and spread, $\Delta N$, from PBHF calculations on pyrrole using STO-3G for different several $\lambda$ combinations and various active spaces. Isolated pyrrole has 36 electrons.

| | | Full space | | 2 active | | 4 active | | 6 active | |
|---|---|---|---|---|---|---|---|---|---|
| $\lambda_{\text{HOMO}}$ | $\lambda_{\text{LUMO}}$ | $\langle N_{el} \rangle$ | $\Delta N$ | $\langle N_{el} \rangle$ | $\Delta N$ | $\langle N_{el} \rangle$ | $\Delta N$ | $\langle N_{el} \rangle$ | $\Delta N$ |
| 1 | 2 | 36.037 | 0.721 | 36.052 | 0.697 | 36.062 | 0.711 | 36.062 | 0.711 |
| 2 | 1 | 35.833 | 0.714 | 35.809 | 0.690 | 35.842 | 0.705 | 35.842 | 0.705 |
| 10 | 20 | 35.997 | 0.729 | 36.001 | 0.707 | 36.001 | 0.729 | 36.008 | 0.730 |
| 20 | 10 | 35.984 | 0.729 | 35.986 | 0.707 | 35.988 | 0.729 | 35.989 | 0.729 |
| 100 | 200 | 35.996 | 0.729 | 36.000 | 0.707 | 36.001 | 0.729 | 36.001 | 0.729 |
| 200 | 100 | 35.995 | 0.729 | 35.999 | 0.707 | 36.000 | 0.729 | 35.996 | 0.730 |

**Table S3:** Average number of electrons, $\langle N_{el} \rangle$, and spread, $\Delta N$, from PBHF calculations on formamide using STO-3G for several $\lambda$ combinations and various active spaces. Isolated formamide has 24 electrons.

| | | Full space | | 2 active | | 4 active | | 6 active | |
|---|---|---|---|---|---|---|---|---|---|
| $\lambda_{\text{HOMO}}$ | $\lambda_{\text{LUMO}}$ | $\langle N_{el} \rangle$ | $\Delta N$ | $\langle N_{el} \rangle$ | $\Delta N$ | $\langle N_{el} \rangle$ | $\Delta N$ | $\langle N_{el} \rangle$ | $\Delta N$ |
| 1 | 2 | 24.093 | 0.706 | 24.112 | 0.691 | 24.110 | 0.695 | 24.110 | 0.695 |
| 2 | 1 | 23.851 | 0.704 | 23.860 | 0.690 | 23.864 | 0.693 | 23.864 | 0.693 |
| 10 | 20 | 23.979 | 0.720 | 24.007 | 0.707 | 23.989 | 0.716 | 23.982 | 0.720 |
| 20 | 10 | 23.965 | 0.720 | 23.987 | 0.707 | 23.975 | 0.716 | 23.968 | 0.719 |
| 100 | 200 | 23.972 | 0.720 | 24.000 | 0.707 | 23.984 | 0.716 | 23.976 | 0.719 |
| 200 | 100 | 23.971 | 0.720 | 23.999 | 0.707 | 23.982 | 0.716 | 23.974 | 0.719 |
